# Adult Neuroplasticity: More Than 40 Years of Research

**DOI:** 10.1155/2014/541870

**Published:** 2014-05-04

**Authors:** Eberhard Fuchs, Gabriele Flügge

**Affiliations:** ^1^German Primate Center, Leibniz Institute for Primate Research, Kellnerweg 4, 37077 Göttingen, Germany; ^2^Department of Neurology, Medical School, University of Göttingen, 37075 Göttingen, Germany

## Abstract

Within the last four decades, our view of the mature vertebrate brain has changed significantly. Today it is generally accepted that the adult brain is far from being fixed. A number of factors such as stress, adrenal and gonadal hormones, neurotransmitters, growth factors, certain drugs, environmental stimulation, learning, and aging change neuronal structures and functions. The processes that these factors may induce are morphological alterations in brain areas, changes in neuron morphology, network alterations including changes in neuronal connectivity, the generation of new neurons (neurogenesis), and neurobiochemical changes. Here we review several aspects of neuroplasticity and discuss the functional implications of the neuroplastic capacities of the adult and differentiated brain with reference to the history of their discovery.

## 1. Introduction

The term “neuronal plasticity” was already used by the “father of neuroscience” Santiago Ramón y Cajal (1852-1934) who described nonpathological changes in the structure of adult brains. The term stimulated a controversial discussion as some neuropathologists favored the “old dogma” that there is a fixed number of neurons in the adult brain that cannot be replaced when the cells die (for review see [1]). In a wider sense, plasticity of the brain can be regarded as “the ability to make adaptive changes related to the structure and function of the nervous system” [2]. Accordingly, “neuronal plasticity” can stand not only for morphological changes in brain areas, for alterations in neuronal networks including changes in neuronal connectivity as well as the generation of new neurons (neurogenesis), but also for neurobiochemical changes. We provide here a short overview of different forms of neuroplasticity with reference to the history of their discovery.

## 2. Changes in Neuron Morphology

In the late 1960s, the term “neuroplasticity” was introduced for morphological changes in neurons of adult brains. Using electron microcopy Raisman [3] demonstrated an “anatomical reorganization” of the neuropil in the septal nuclei of adult rats after a selective lesion to distinct axons which terminate on the neurons in those nuclei. Since then, many changes in the morphology of neurons in response to various internal and external stimuli have been described. A strong external stimulus that evokes numerous neuroplastic changes is stress. Repeated or chronic stress changes the morphology of neurons in various brain areas. Probably the most thoroughly investigated neuromorphological change is the stress-induced regression of the geometrical length of apical dendrites of pyramidal neurons that was first demonstrated in the hippocampus [4]. The hippocampus is part of the limbic-HPA (hypothalamic-pituitary-adrenal) system and regulates the stress response. Retraction of dendrites of CA3 pyramidal neurons has been repeatedly documented after chronic stress as well as after chronic glucocorticoid administration [5–7]. Dendritic retraction does of course reduce the surface of the neurons which diminishes the number of synapses. Also neurons in the medial prefrontal cortex retract their dendrites in response to stress, but the effects depend on the hemisphere [8, 9]. Studies on the prefrontal cortex showed that neurons in this brain region are particularly plastic in that they change their dendritic morphology with the diurnal rhythm [10]. Such neuroplastic reactions are not a one-way road. In the amygdala, the dendritic arborization of the pyramidal and stellate neurons in the basolateral complex was* enhanced* by a similar chronic stress paradigm that reduces branching of dendrites in hippocampal CA3 pyramidal neurons [11]. The brain's pronounced neuroplastic capacities are also reflected by the fact that the synapses are replaced as soon as the stress is terminated [12]. Furthermore, drugs that stimulate neuroplasticity can prevent the stress-induced retraction of dendrites in the hippocampal formation [13]. A form of functional neuroplasticity is long-term potentiation (LTP), that is the long-lasting enhancement in signal transmission between two neurons after synchronous stimulation [14].

## 3. Neuron Death

The research on neuroplasticity in adult brains was strongly stimulated by observations that brain neurons may die, for example, because of trauma or degenerative illnesses such as Parkinson's or Alzheimer's disease [15]. In the late 1990s, there were reports that even the stress that an individual experiences can kill neurons in the brain. This message was based on studies in wild vervet monkeys that had been housed in a primate center in Kenya where they died suddenly. The animals had experienced severe stress because of social isolation from their group [16]. The finding that their brains revealed dead pyramidal neurons in the hippocampus attracted great public attention as the message was reduced to “stress kills neurons.” However, it later turned out that in this study on wild life animals the* post mortem* treatment of the brain tissue had been not optimal. The time between death of the animals and fixation of the brains for the neuropathological analysis was obviously too long so that morphology of the neurons was affected to an extent that had nothing to do with the previous stress exposure of the living animals. Since stress raises plasma glucocorticoids (GC), monkeys were chronically treated with GC in a subsequent study, and also the brains of these animals revealed changes in neuron morphology that were interpreted as dead or dying neurons [17]. However, these findings could not be confirmed by others. Instead, it was recognized that the morphological analysis of pyramidal neurons is technically delicate. It became apparent that, after a subject's death, neurons may dramatically change their morphology and turn into “dark neurons” when the brain tissue has not been fixed adequately for the histological analysis [18]. When the chronic stress experiments were repeated under conditions that acknowledged those technical issues, it turned out that stress* does not* kill neurons, which is definitely a good message for stressed individuals [19]. Further studies showed that apoptosis (programmed cell death) in the hippocampal formation is a relatively rare event and that chronic stress may even reduce cell death in certain hippocampal subfields while increasing apoptosis in others [20]. Since chronic social stress in animals is regarded as preclinical model for depression the finding of a lack of neuron death in stressed animals also shed new light on a hypothesis saying that, in humans, major depression kills neurons in the brain. Indeed, it was later found that hippocampal neuron numbers in depressed subjects do not significantly differ from the numbers in healthy individuals [21]. Also the hypothesis that chronic GC exposure leads to neuron death had to be revised. A summary of a range of studies on these issues concluded that it is unlikely that endogenous GC can cause structural damage to the hippocampal formation [22]. Nevertheless it is an established fact that “adverse influences” such as stress, depression, and chronic GC treatments may cause shrinkage of the hippocampal formation [23]. However, the underlying processes are obviously not neuron loss but other changes in the tissue such as reductions in neuronal dendrites and further presumptive alterations in the neuropil that have not been identified in detail yet ([6, 24]; for review see [25]).

## 4. Neurogenesis in Adult Vertebrates

The most appealing phenomenon of neuroplasticity appears to be adult neurogenesis, that is the generation of new neurons in adult brains. Neurogenesis takes of course place in the developing central nervous system, but in view of the fact that certain illnesses such as Parkinson's disease and multiple sclerosis occur in adulthood the interesting question is whether also adult brains are able to replace lost neurons.

In contrast to most cells of the body such as those in the gut, the skin, or the blood which are constantly renewed, the brain—and in particular the mammalian brain—has always been regarded as a nonrenewable organ. Most neurons of the adult central nervous system appear as terminally differentiated. Although the adult brain can sometimes functionally compensate for damage by generating new connections among surviving neurons, it does not have a large capacity to repair itself because most brain regions are devoid of stem cells that are necessary for neuronal regeneration. This lack of neuroplasticity was first described by Santiago Ramón y Cajal who stated that “In adult centers the nerve paths are something fixed, ended, immutable. Everything may die, nothing may be regenerated. It is for science of the future to change, if possible, this harsh decree” [26].

The “no new neurons” dogma was already challenged almost five decades ago. Using autoradiography with the tritiated DNA nucleoside ^3^H-thymidine, Altman [27, 28] gained first evidence for the production of glia cells and possibly also of neurons in the brains of young adult rats and adult cats. In subsequent studies, 10-day-old rats received ^3^H-thymidine and the tritium radioactivity was visualized 2 months later in cells of the subgranular zone in the dentate gyrus [29]. Unfortunately, autoradiography with ^3^H-thymidine is a very delicate method and it is not easy to pick up the low number of neurons that is generated daily in, for example, the dentate gyrus of adult mammals. Accordingly, ^3^H-thymidine autoradiographs produced at that time could not generally convince the scientific community that adult neurogenesis really exists. Thus only a limited number of experiments followed the initial studies mentioned above. However, the neuronal character of newly generated cells in the rodent dentate gyrus was confirmed and further substantiated by demonstrating that these newborn cells receive synaptic input and extend axons into the mossy fiber pathway that projects to the CA3 subfield [30–32]. Another landmark was in the early 1980s, when substantial neurogenesis was demonstrated in a vocal control nucleus of the adult canary brain [33], and a functional link between behavior, song learning, and the production of new neurons was established [34]. The finding that, in songbirds (canaries, zebra finches), males have larger song control nuclei in their brains as compared to females indicated that the number of neurons in those adult birds may change with the season [35]. Indeed, the neuron number in song control nuclei increases in spring time when male zebra finches begin to sing, and newborn neurons were also found in the HVC (hyperstriatum ventrale, pars caudalis) of adult canaries [36]. Studies on the HVC in birds showed that steroid hormones play important roles in these processes of neuroplasticity, in particular the gonadal hormone testosterone [35, 37].

**Figure 1:**
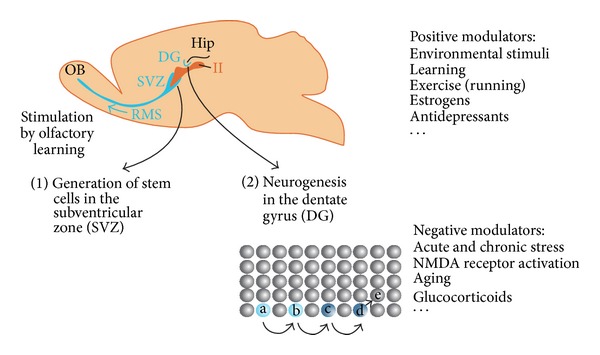
A schematic view on adult neurogenesis. Neuronal progenitor cells are generated in the subventricular zone (SVZ) and in the dentate gyrus (DG) of the hippocampal formation (Hip). (1) In the SVZ, neuroepithelial progenitor cells are generated that migrate through the RMS (rostral migratory stream) to the olfactory bulb (OB). They differentiate to mature neurons and are integrated as functional elements into the neuronal olfactory circuitry. (2) In the DG, quiescent neural progenitors (a) become amplifying neural progenitors (b) that differentiate first to neuroblasts (c), then to immature neurons (d), and finally to functionally mature granule neurons (e).

In line with these findings Cajal's statement on the fixed number of neurons in adult brains was further challenged as it became clear that even in mammals, parts of the adult central nervous system are able to replace neurons. In the olfactory epithelium of the mammalian nose, sensory neurons are continuously generated throughout the lifespan, as first shown in adult squirrel monkeys [38]. This electron microscopic study clearly showed large numbers of newborn sensory neurons that are produced every day in the olfactory epithelium of the adult animals. Later it was found that also neurons in the olfactory bulb (OB) of adult mammals can be replaced. The new OB neurons derive from the subventricular zone at the lateral ventricle where neuroblasts are generated that migrate through the rostral migratory stream to the OB (Figure 1). The neuroblasts differentiate to functional neurons, in that case granule cells, which form synapses with mitral cells ([39, 40]; for review see [41]). However, OB neurogenesis is easier to detect than hippocampal neurogenesis and it took several years until there was reliable evidence that hippocampal neurogenesis does exist in adult mammals.

In particular, neurogenesis could long not be demonstrated in the brains of adult nonhuman primates such as rhesus monkeys thereby leading to the assumption that neuronal replication is not tolerated in primates. In an initial study, Rakic [42] investigated neurogenesis in adult rhesus monkeys using ^3^H-thymidine, examining major structures and subdivisions of the brain including the visual, motor, and the association neocortex, hippocampus and OB. Rakic found “not a single heavily labeled cell with the morphological characteristics of a neuron in any brain in any adult animal” and concluded that “all neurons of the rhesus monkey brain are generated during prenatal and early postnatal life” [42, 43]. Furthermore, Rakic argued that “a stable population of neurons may be a biological necessity in an organism whose survival relies on learned behavior acquired over a long period of time.” These statements had a profound influence on the development of the research field in that they formed the basis for researchers of the time to show little interest to detect neurogenesis in the adult mammalian brain.

A revolution in the field of neurogenesis research took place when the thymidine analog 5-bromo-2′-deoxyuridine (BrdU) and corresponding antibodies were introduced for labeling newborn neurons by immunohistochemistry [44]. Using this new—and in comparison to autoradiography—simple and fast technique, it became clear that adult hippocampal neurogenesis in mammals is not restricted to rodents but has been conserved throughout mammalian evolution. The formation of new granule neurons was, for example, demonstrated in the dentate gyrus of adult rats and tree shrews [45, 46]; the later species is regarded as phylogenetically located between insectivores and primates [47]. Evidence of neurogenesis in the adult primate brain derived from studies in marmoset monkeys [48], a small nonhuman primate from South America, and in macaques which are typical representatives of the nonhuman Old-World primates [49, 50]. Finally, the existence of neurogenesis in the adult human brain was shown in cancer patients who were injected with BrdU to monitor tumor cell proliferation. Some of these patients died from their illness and small samples of their hippocampi were evaluated for the presence of BrdU-labeled neurons. Since BrdU had been systemically administered, all dividing cells were supposed to be labeled. Indeed, newborn neurons were detected in the dentate gyrus granule cell layer of all individuals [51]. These data unequivocally showed that adult neurogenesis is a common phenomenon across mammalian species. It thus became generally accepted that adult neurogenesis not only does occur in the olfactory bulb and the* gyrus dentatus* of the hippocampal formation of mammals but can also be detected in “higher” brain regions such as the neocortex [52, 53]. However, there are still open questions regarding the extent of neurogenesis in homologous brain regions of different mammalian species (see below).

To detect neurogenesis in brains of adult humans the group of J. Frisén took advantage of the increased concentration of ^14^C in the atmosphere after nuclear bomb tests [54]. After a nuclear explosion, this radioisotope is increasingly incorporated into dividing cells of living organisms, including humans. Through the determination of ^14^C, the authors found that about 700 new neurons are generated daily in the hippocampal formation of adult humans. Interestingly, the ^14^C analysis of human brains revealed adult neurogenesis in the striatum, adjacent to a site at the lateral ventricle where neuronal precursor cells are generated, and there are indications that the neuroblasts in the human striatum differentiate to interneurons [55]. Surprisingly, no newborn neurons could be detected with the ^14^C technique in the adult human OB. These most recent findings clearly show that species and brain-region specific processes of neurogenesis await further elucidation.

Adult neurogenesis does occur not only in mammals and birds but also in amphibians, reptiles, and bony fishes (for references see [56]). Despite this omnipresence of adult neurogenesis within vertebrates, comparative studies have revealed significant differences between classes. So far it appears that in most mammals, the generation of new neurons in adult brains takes place in two regions, the subventricular zone and the dentate gyrus, and the number of newly generated neurons is small compared to the total number of brain cells (Figure 1). However, there are also reports from studies in mice that new neurons can be generated in the adult substantia nigra, although with “a slow physiological turnover of neurons” [57]. In contrast, in fish a huge number of neurons are continuously produced in many areas of the adult brain [56]. Also important to mention that in comparison with fishes, reptiles and birds, the rate of neurogenesis in adult mammals decreases with age [58].

## 5. Regulators of Adult Neurogenesis

The existence of neurogenesis in adult brains gives hope that even damaged brain regions can be functionally repaired. Indeed, injury to the adult brain such as ischemic insults stimulates the proliferation of subventricular zone cells and thus the formation of neuronal precursor cells. These neuroblasts migrate along blood vessels to the damaged region (for review see [41]). However, only a small percentage can survive, in part because inflammatory processes that occur in the ischemic brain region inhibit neurogenesis and the successful integration of new cells into a functional neuronal network [59]. Anti-inflammatory drugs can restore neurogenesis, as shown in rodent models of peripheral inflammation and after irradiation [60].

Knowledge about the regulation of adult neurogenesis is definitely a prerequisite for future therapeutic interventions that may take advantage of the generation of new neurons in adult brains. Kempermann [61] emphasized that there is an “immense spectrum of neurogenic regulators” which reflect “the sensitivity of adult neurogenesis to many different types of stimuli.” Respective regulatory elements that are so far known include single molecules as well as environmental conditions that lead to changes in a large number of factors which themselves influence neurogenesis. Among the molecular factors that were first identified as regulators of adult neurogenesis are sex steroids such as estrogen which can at least transiently stimulate neurogenesis in the dentate gyrus [62]. Steroid hormones have pleiotropic effects on the expression of many genes among which are also genes which themselves encode regulators of neurogenesis. Accordingly, in female mammals, effects of steroid hormones on adult neurogenesis depend on the estrous cycle and other stages related to reproductive biology [63]. It is not surprising that growth factors such as BDNF (brain-derived neurotrophic factor) and VEGF (peripheral vascular endothelial growth factor) regulate adult neurogenesis [64–66]. Also the neurotransmitter glutamate and astroglia have an impact on adult neurogenesis, probably by generating a distinct microenvironment that may favor the generation/differentiation of neuroblasts [67–69]. The large number of factors that regulate adult neurogenesis has been reviewed before [70].

Effects of stress on neurogenesis in the dentate gyrus (the so-called hippocampal neurogenesis) have been studied by several groups. Chronic social stress in tree shrews and other adverse stress experiences in marmoset monkeys reduced hippocampal neurogenesis [23, 46, 48]. The effects of social and other forms of stress depend on the stressor's intensity and its duration, and they may be reversible [71]. Prenatal stress in rhesus monkeys has persistent effects as a reduction in neurogenesis was observed in the adolescent individuals [72]. In newborn marmoset monkeys which were intrauterinely exposed to the synthetic glucocorticoid dexamethasone, the proliferation of putative precursor cells but not the differentiation into mature cells was impaired [73]. Interestingly, this decreased proliferation rate observed in newborn monkeys was no longer detectable in their 2-year-old siblings suggesting no long-lasting effect of prenatal hyperexposure to dexamethasone on neuronal proliferation and differentiation in the dentate gyrus of marmoset monkeys [74].

Several authors attributed the effects of stress on neurogenesis to the actions of glucocorticoids which are elevated in the blood of stressed individuals. Corticosteroids do indeed regulate neurogenesis and the glucocorticoid receptor antagonist mifepristone prevented the stress-induced reduction in hippocampal neurogenesis [75]. Also the mineralocorticoid receptor appears to play a particular role as indicated by the fact that a genetic disruption of the receptor impaired adult hippocampal neurogenesis in mice [76]. However, elements of the glucocorticoid system are not the only regulatory factors of adult neurogenesis in stress. Instead, as pointed out above, other components of the stress cascade such as enhanced excitatory neurotransmission (increased glutamate release) play also a role. In several preclinical models of depression using stress to induce depressive-like symptoms in animals, certain antidepressants restored the neurogenesis that had been impaired by the stress (see, e.g., [23, 77]). There are indications that antidepressants activate the glucocorticoid receptor which may increase hippocampal neurogenesis [78]. However, it remains an enigma whether endogenous or synthetic substances exist that can boost adult neurogenesis via this receptor system.

The formation of new neurons is regulated by substances derived from blood vessels and is targeted by an enormous number of factors [61, 79]. Coinciding with this view are reports demonstrating that adult neurogenesis is enhanced by physical activity such as running [80], by learning [81], or by environmental enrichment [82–84].

## 6. Functional Role of Adult Neurogenesis

Soon after the discovery of adult neurogenesis it was hypothesized that hippocampal neurogenesis (i.e., the neurogenesis in the subgranular zone of the dentate gyrus, a region of the hippocampal formation) plays a crucial role in learning and memory [81]. However, experimental results on the role of different forms of memory in adult rodents (e.g., spatial learning versus associative memory) were in part contradictory. In a comprehensive review, Koehl and Abrous [85] came to the conclusion that adult neurogenesis in rodents is involved “when the task requires the establishment of relationships among multiple environmental cues…for the flexible use of acquired information.” Whether this is true for all mammals remains to be determined as a low rate or even absence of neurogenesis was found in the hippocampal formation of adult bats [86] and in whales [87], species with an excellent spatial working memory. In the OB, adult-born new neurons are integrated into the neuronal circuits that are responsible for olfaction and olfactory memory, respectively (for review see [88]).

The fact that in animal models of depression certain antidepressants restored normal neurogenesis that had been impaired by stress led to the hypothesis that the beneficial effects of antidepressants depend on the restoration of normal neurogenesis [77]. The volume of the hippocampal formation is reduced in patients with major depression, and antidepressants can normalize hippocampal volume [89]. However, the hippocampal shrinkage is probably not due to a decrease in neurogenesis but rather to more complex changes in the neural network which involve dendritic, axonal, and possibly also glial alterations [24]. Kempermann et al. [90] proposed that “failing adult hippocampal neurogenesis may not explain major depression, addiction or schizophrenia, but contributes to the hippocampal aspects of the diseases.” A comparison of the neural stem-cell proliferation in* post mortem* brain samples from patients with major depression, bipolar affective disorder, schizophrenia, and control subjects revealed no evidence of reduced neurogenesis in the dentate gyrus of depressed individuals. Furthermore, antidepressant treatment did not increase neural stem-cell proliferation. Unexpectedly, significantly reduced numbers of newly formed cells were found only in schizophrenic patients [91]. Concerning impaired neurogenesis as presumptive cause of depression a group of experts summarized that “a lasting reduction in neurogenesis” … (is) “unlikely to produce the full mood disorder” [92]. However, more recent reports based on* post mortem* studies showed decreased numbers of neuronal progenitor cells in the dentate gyrus of depressed patients and a selective enhancing effect of antidepressant treatment in the anterior and middle dentate gyrus of depressed individuals [93–95]. To overcome the manifold limitations of* post mortem* studies, a future approach to address the question of adult neurogenesis in humans more precisely (possibly in longitudinal studies) could be the visualization of this process in live subjects using advanced* in vivo* imaging techniques. Moreover, this approach could help answer the open questions on the role of neurogenesis in cognitive functions and its functional impact and contribution to the etiology of depression.

## 7. Chromatin Changes

**Figure 2:**
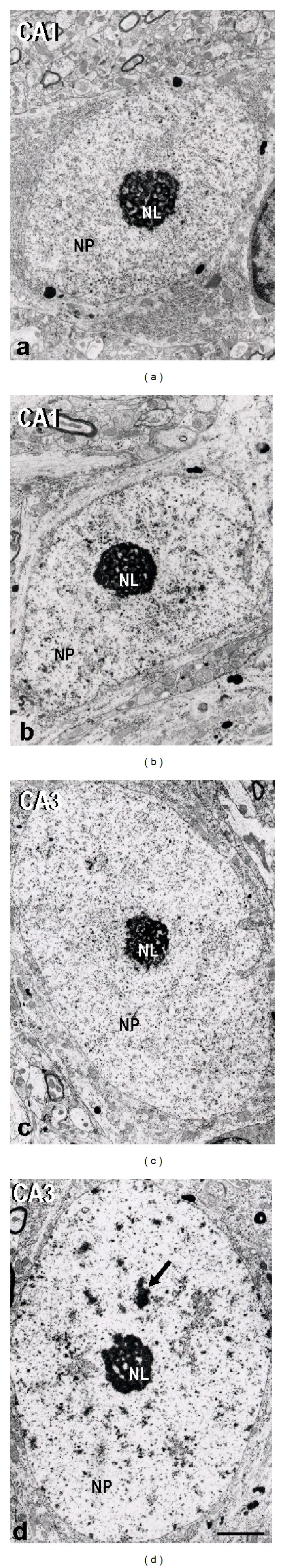
Electron micrographs of pyramidal neuron nuclei in the hippocampus of control and stressed male tree shrews: (a), control CA1; (b), stress CA1; (c), control CA3; (d), stress CA3. Note the homogeneous nucleoplasma (NP) in the controls and the large number of heterochromatin clusters (arrow) in nuclei of CA3 pyramidal neurons in stressed animals. NL: nucleolus. Calibration bar: 2 *μ*m.

**Figure 3:**
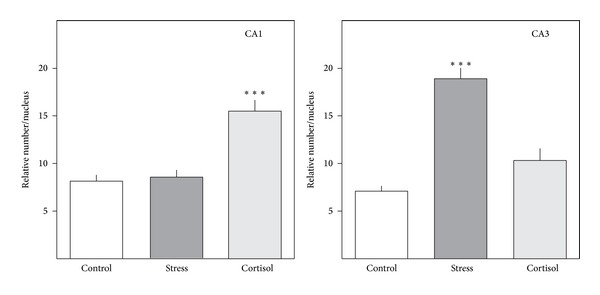
Relative number of heterochromatin clusters in electron micrographs from nuclei of pyramidal cells in CA1 and CA3. Data are mean ± SEM (*P* ≤ 0.0001).

When searching for dead neurons in the hippocampal formation of male tree shrews, standard histology showed that chronic social stress does not lead to neuronal death but changes the appearance of the nuclei in the hippocampal neurons [96]. Closer investigations revealed that chronic stress increases the formation of heterochromatin in the nuclei of the hippocampal neurons [97]. In this study, the nuclear ultrastructure of hippocampal pyramidal neurons in male tree shrews that had been exposed to daily social stress during four weeks according to a standard stress paradigm was analyzed. Electron microscopic analysis revealed that in the stressed animals the nucleoplasma of CA3 pyramidal neurons displayed numerous heterochromatin clusters (Figure 2). Heterochromatin is a form of condensed chromatin whose occurrence indicates that transcription of genes is reduced in those cells. Quantification of the clusters revealing areas larger than 1 *μ*m² in the hippocampal region CA3 showed that there was more heterochromatin in stressed animals compared to controls. In contrast, in area CA1, the stress had no effect on the density of heterochromatin clusters (Figure 3; [97]). Although in those days it was totally unknown which genes in the hippocampal nuclei were “silenced” by the chronic stress, these morphological data indicated already what was later called “epigenetics,” the phenomenon that environmental factors change the structure of chromatin, influence transcription, and induce changes in the genome [98]. Since glucocorticoid hormones are often regarded as important factors that convey many effects of chronic stress, it was tested whether a chronic cortisol treatment would have the same effects on the chromatin as the chronic social stress. Interestingly, chronic cortisol changed the number of heterochromatin clusters only in hippocampal region CA1, but not in CA3, the region that is targeted by stress (Figure 3). These results indicate a site and treatment specific reaction to stress and glucocorticoid treatment in the hippocampal formation. The obvious differences between chronic stress and chronic glucocorticoid treatment must be kept in mind because they possibly reflect different cellular pathways activated by the two treatments.
